# Granulomatous cheilitis of Miescher: a rare entity

**DOI:** 10.1186/s12905-023-02280-9

**Published:** 2023-03-21

**Authors:** Santosh Tummidi, Prabhakaran Nagendran, Michael Leonard Anthony, Rupa Jami Ramani, Arundhathi Shankaralingappa, Hima Gopinath

**Affiliations:** 1 Department of Pathology & Lab Medicine, AIIMS, Kalyani, India; 2 Department of Dermatology, AIIMS, Mangalagiri, India; 3 Department of Pathology, AIIMS, Mangalagiri, India

**Keywords:** Cheilitis, Case report, Lip, Recurrence, Steroids, Female

## Abstract

**Background:**

Granulomatous cheilitis (GC) is a rare entity of unknown etiology. It is a chronic inflammatory disorder with a predilection for young females. It is characterized by asymptomatic unrelenting swelling of lips. It is a monosymptomatic form or an incomplete variant of Melkersson-Rosenthal syndrome (a triad of recurrent orofacial swelling, relapsing facial paralysis, and fissuring of the tongue).

**Case presentation:**

We herewith report a case of a 27-year-old female, presenting with persistent upper lip swelling, for 3 months. Biopsy from the lesion revealed features of granulomatous cheilitis.

**Conclusion:**

GC should be considered in the differential diagnosis of unrelenting swelling in the lip. Spontaneous remission is rare, and recurrences are common. Corticosteroids used for treatment provide temporary improvement.

## Background

Granulomatous cheilitis (GC), also called Miescher cheilitis, was first described by Miescher in 1945, as a rare idiopathic inflammatory disorder. It’s characterized by chronic persistent, painless swelling of the lips with noncaseating granulomatous infiltration [1]. It has been linked to orofacial granulomatosis (OFG) as well as Melkersson-Rosenthal syndrome (MRS) [1, 2].

Wiesenfeld et al*.* had first described OFG as idiopathic persistent and/or recurrent labial enlargement, oral ulcers, and a spectrum of orofacial features with the absence of any other identifiable systemic conditions [3]. Melkersson (1928) had identified the association of relapsing facial palsy and transient facial edema with GC [4]. The addition of fissured tongue (Rosenthal, 1931) completed the triad of recurrent orofacial edema, relapsing facial nerve palsy, and fissuring of the tongue (lingua plicata) which later became eponymous with Rosenthal and Melkersson [4].

GC also continues to be regarded as a monosymptomatic form of MRS ever since such first reference by Martin 1959 [2]. However, a possibility of GC and MRS being separate diseases too exists. We report a case of monosymptomatic GC in a 27-year-old female.

## Case presentation

A 27-year female presented to our Dermatology clinic, with a 3 months history of unrelenting upper lip swelling. The swelling gradually increased in size to involve the whole lip. She had no history of local trauma, atopy, or applied irritants and worked in a gold shop. She also reported burning and peeling off in upper lip. History revealed that she had been treated with oral antihistamines which showed partial response but later became unresponsive.

**Fig. 1:**
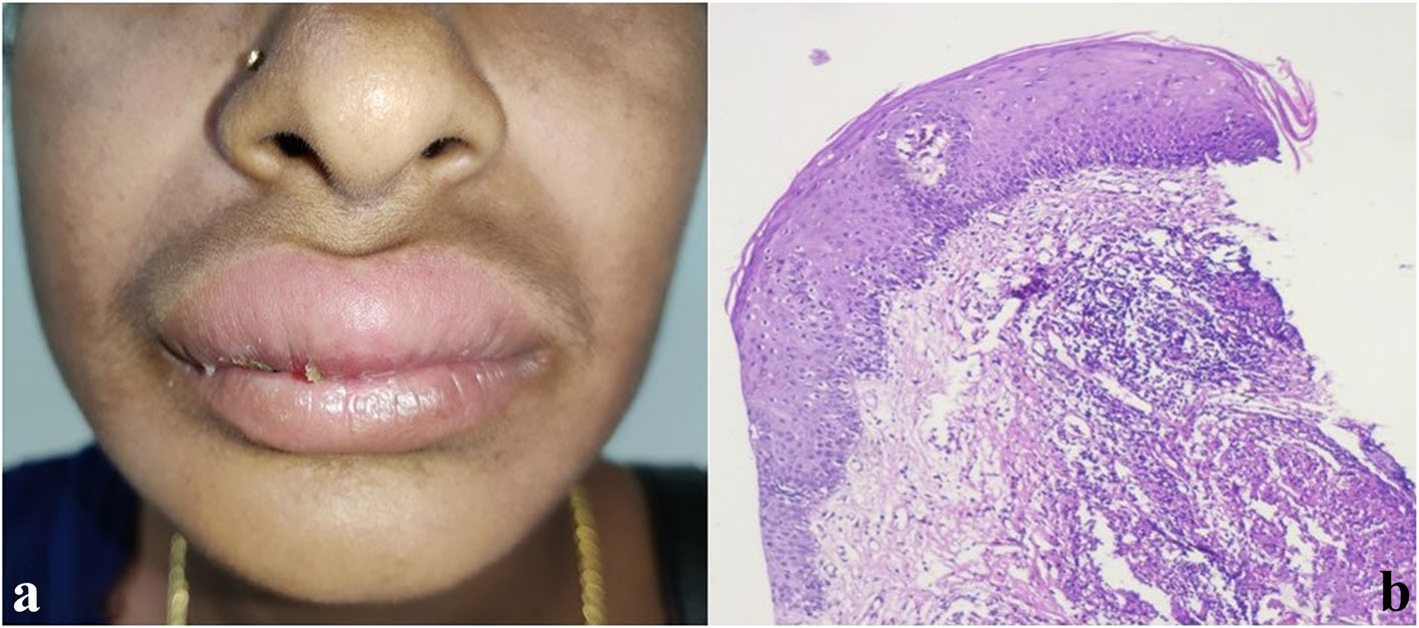
**a** Swelling of upper lip and perioral area, diffusely swollen, firm, non-tender, with areas of scaling. **b** Microscopy showing mucosal lining with parakeratotic stratified squamous epithelium. Mid dermis with presence of non-caseating granuloma. (H&E, × 10)

On examination, her upper lip and the perioral area were diffusely swollen, firm, and non-tender, with areas of scaling in the upper lip [Fig. 1a]. The tongue was normal-appearing and there was no associated regional lymphadenopathy. Facial nerve examination was also normal. There was no history of fever, gastrointestinal symptoms, fatigue, or weight loss. Her stool for occult blood was negative. No known drug/food allergy was recorded from the patient.

Her complete blood counts were within normal limits. Her serum calcium (9.1gm/dl) and ACE (Angiotensin-converting enzyme) were within normal limits. Chest radiography and high-resolution computed tomography scan of the lungs were normal and did not show any hilar lymphadenopathy.

**Fig. 2:**
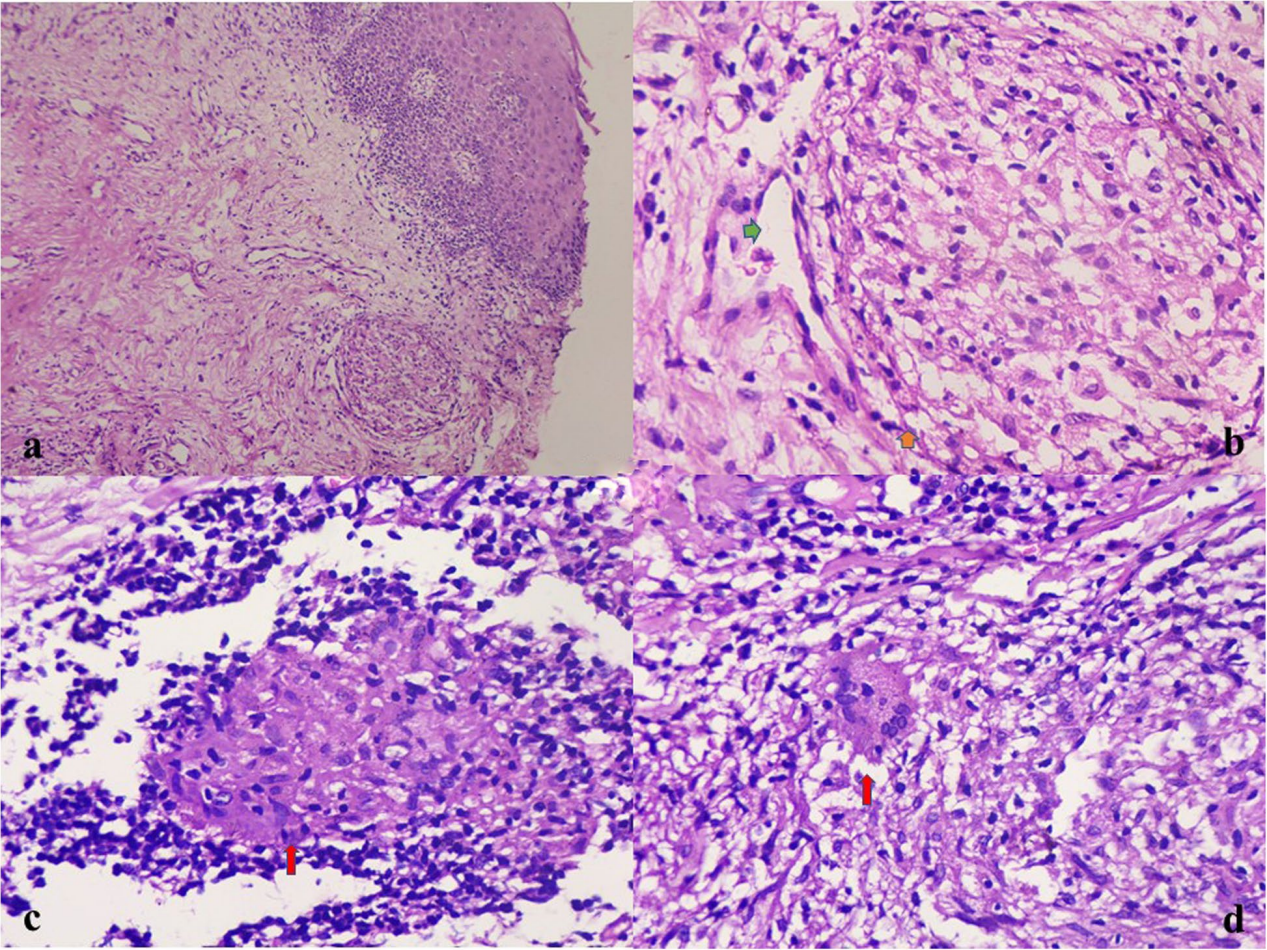
**a, b, c, d** Microscopy showing mucosal lining with parakeratotic stratified squamous epithelium. Mild papillary dermal edema. Mid & deep dermis with presence of non-caseating granuloma comprising of epithelioid cells, with sprinkling of lymphocytes, histocytes (orange arrow Fig. 2b), multinucleated giant cells (red arrow Fig. 2d) and occasional plasma cells was seen. Few dilated lymphatics (green arrow Fig. 2b) were also seen adjacent to granuloma along with mild perivascular lymphoplasmacytic cuffing. (H&E, x 20 & × 40)

**Fig. 3:**
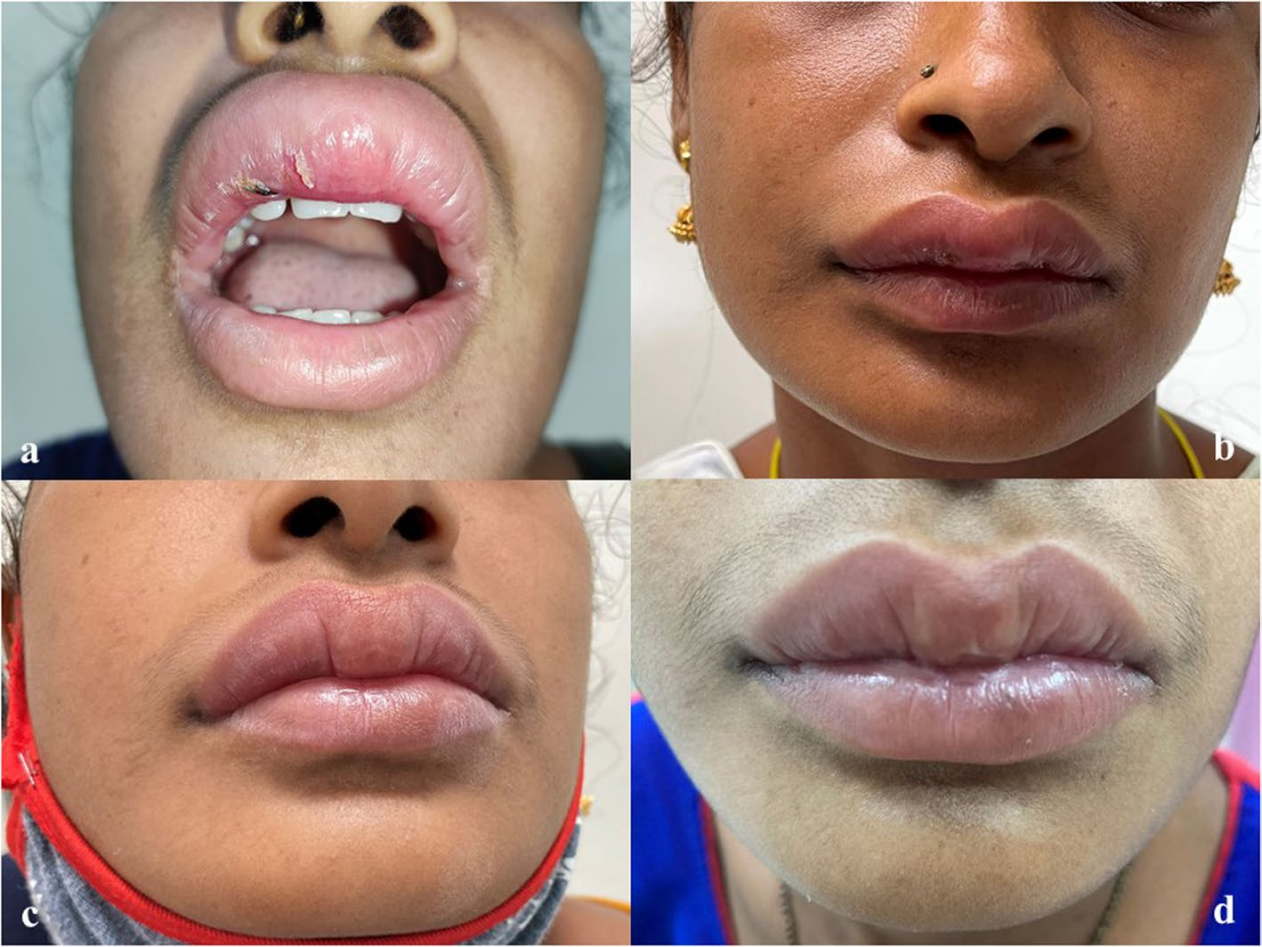
**a:** Presentation with swelling upper lip; **b:** Follow up of patient at one month; **c:** Follow up of patient at two months; **d:** Follow up of patient at three months

A biopsy from the upper lip was taken. A skin-covered soft tissue piece was received measuring approximately 0.5 × 0.5 cm. Microscopy revealed epidermis with parakeratotic stratified squamous epithelium, variable acanthosis, mild spongiosis, irregular rete ridges, with lymphocytic exocytosis. Mild to moderate dermal edema and presence of non-caseating granuloma comprising epithelioid cells, with a sprinkling of lymphocytes, histocytes, multinucleated giant cells, and occasional plasma cells were seen. Few dilated lymphatics were also seen adjacent to granuloma along with mild perivascular lymphoplasmacytic cuffing [Fig. 1b and 2a-d]. Ziehl Neelsen (ZN) stain for acid-fast bacilli and Periodic acid Schiff (PAS) for fungal elements were negative. Mantoux test was negative. A diagnosis of Granulomatous cheilitis was made. She was administrated intralesional triamcinolone acetonide at 10 mg/ml. 4 sittings of intralesional steroid were given with 3 weeks interval between each sitting. On follow up there is a nearly 40% reduction in the lesion [Fig. 3a-d]. Beyond that there was no response and the lesion resumed to its original size in another 4 months. After that patient was started on azithromycin pulse therapy (weekly azithromycin 500 mg OD for three consecutive days) for a month with no improvement. Then the patient defaulted for about 6 months. When the patient revisited we started intralesional triamcinolone at a higher dose (40 mg/ml) and oral minocycline 100 mg once daily. We gave three doses of intralesional steroids and continuing oral minocycline. Now patient showed nearly 80% improvement and she is on follow up.

## Discussion

GC is a granulomatous condition, manifesting as chronic recurrent swelling of one or both the lips. GC associated with facial muscle palsy and fissured tongue is known as Melkerson-Rosenthal syndrome (MRS) [5]. MRS is a rare childhood or adolescent onset neurological disorder of unknown etiology. Both GC and MRS are considered subsets of Orofacial granulomatosis (OFG), introduced by Wiesenfield in 1985 [3]. OFG is a syndrome, grouping non infectious and non-necrotising granulomatous involvement of the lips, oral cavity, and face in addition to the GC, sarcoidosis, and Crohn’s disease [3].

The etiology of GC is still unknown. Several postulated factors include, a) Genetic Chromosome 9p11, b) Immunologic factors—GC is characterized by T helper 1 mediated immune response, c) Allergic factors—allergy to dental materials, foodstuff, food additives, [Table 1] d) Hypersensitivity to UV-B radiation, e) as part of Crohn disease [6–8].

**Table 1:** List of possible agents causing GC

Type	Agents
Atopic reactions	Allergens (Gold, mercury, cobalt, zinc, Gluten rich diet, carbonated drinks, chocolate, cinnamon, beer, food additives, dental amalgam, monosodium glutamate, carmoisine, toothpaste
Drugs	ACE inhibitors, Calcium channel blockers
Granulomatous	Foreign body reactions, sarcoidosis
Hereditary	Angioedema secondary to C1 esterase deficiency and Ascher’s syndrome
Infectious	Tuberculosis, leprosy, histoplasmosis, leishmaniasis, Pepto Streptococcus spp., Eubacterium spp., Streptococcus spp.
Inflammatory	Rosacea
Autoimmune	Crohn’s disease

The incidence of GC is estimated to be 0.08% in the general population. Clinically the 1^st^ episode subsides in hours to days [9]. The upper lip, lower lip, or both lips can be involved. The face, oral mucosa, gums, tongue, pharynx, and larynx can be other sites to be involved. Patients may complain of pain, erythema, fissures, erosions, scaling of lips, and edema [8, 9]. Any age group can be involved, but most commonly seen in adults with a peak incidence in 20–40 years of age with female predominance [8].

Histopathology can reveal normal keratinizing squamous epithelium, and noncaseating granulomatous inflammation in the deeper subcutaneous and parafollicular tissues. Ziehl–Neelsen, silver, Periodic acid-Schiff, and Warthin–Starry stains can be negative for acid-fast (Mycobacteria and Actinomyces, specifically), fungal, and spirochaetal organisms. Associated local lymphadenopathy may be present [5, 10].

MRS is a rare neuro-mucocutaneous syndrome with an apprx. incidenc of 0.08%. Its onset is in 2-3^rd^ decade of life. Presence of all the three features i.e. CG, facial palsy & plicated tongue may be seen in 8- 25% cases. The diffrential diagnosis can be heterogenous disorders ruling out foreign body reaction, sarcoidosis, Crohn’s disease, Wegener’s vasculitis, amyloidosis, orofacial herpes, rosacea [8, 11].

Intralesional corticosteroids with or without immunomodulators are the first line of treatment followed by oral corticosteroids with possible relapse. The second line can include infliximab, clofazimine, metronidazole, and minocycline. In severe cases, cheiloplasty & radiotherapy can be considered [11, 12]. Azithromycin pulse therapy has also been tried in a few cases [13].

## Conclusion

Patients can have a variable clinical presentation of GC and diagnosis is by the exclusion of other granulomatous lesions with similar clinical and histopathological features. Intralesional corticosteroids along with a combination of antibiotics can be beneficial, with regular follow-up.

## Data Availability

All the data regarding the findings are available within the manuscript.

## References

[1] Miescher G (1945). Uber essentielle granulomatose makrocheilie (Cheilitis granulomatosa). Dermatologica.

[2] Sharma YK, Chauhan S, Deo K, Agrawal P (2020). Granulomatous cheilitis: Report of three cases and systematic review of cases and case series reported from India. Clin Dermatol Rev.

[3] Wiesenfeld D, Ferguson MM, Mitchell DN, MacDonald DG, Scully C, Cochran K (1985). Oro-facial granulomatosis – A clinical and pathological analysis. Q J Med.

[4] Greene RM, Rogers RS (1989). Melkersson-Rosenthal syndrome: A review of 36 patients. J Am Acad Dermatol.

[5] Critchlow WA, Chang D (2014). Cheilitis granulomatosa: a review. Head & neck pathol.

[6] Nair PA, Patel TM (2017). Granulomatous cheilitis involving the lower lip. Egypt J Dermatol Venereol.

[7] Shanmukhappa AG, Shivaram B, Budamakuntala L, Samynathan A (2017). Idiopathic granulomatous cheilitis of Miescher in a young patient: A rare entity and its successful treatment. Indian J Paediatr Dermatol.

[8] Jamil RT, Agrawal M, Gharbi A, et al. Cheilitis Granulomatosa. [Updated 2020 Nov 30]. In: StatPearls. Treasure Island (FL): StatPearls Publishing; 2021. Available from: https://www.ncbi.nlm.nih.gov/books/NBK470396/29261927

[9] Chintagunta SR, Sana SN, Bukka KS, Padma S (2017). Cheilitis granulomatosa: Case series. J NTR Univ Health Sci.

[10] Nair SP (2016). Cheilitis granulomatosa. Indian. Dermatol Online J.

[11] Tambe S, Patil P, Modi A, Jerajani H (2018). Metronidazole as a monotherapy in the management of granulomatous cheilitis. Indian J Dermatol Venereol Leprol.

[12] Vibhute NA, Vibhute AH, Daule NR (2013). Cheilitis granulomatosa: A case report with review of literature. Indian J Dermatol.

[13] Bruett Carter T, Trump Bryan R, Adams David R, Halpern Leslie R (2020). Orofacial granulomatosis: A case treated with azithromycin pulse therapy, review of the literature and an algorithm for diagnosis. IDCases.

